# One Shoot, Two Birds: Alleviating Inflammation Caused by Ischemia/Reperfusion Injury to Reduce the Recurrence of Hepatocellular Carcinoma

**DOI:** 10.3389/fimmu.2022.879552

**Published:** 2022-05-11

**Authors:** Hao Chen, Di Lu, Xinyu Yang, Zhihang Hu, Chiyu He, Huigang Li, Zuyuan Lin, Modan Yang, Xiao Xu

**Affiliations:** ^1^ Key Laboratory of Integrated Oncology and Intelligent Medicine of Zhejiang Province, Department of Hepatobiliary and Pancreatic Surgery, Affiliated Hangzhou First People’s Hospital, Zhejiang University School of Medicine, Hangzhou, China; ^2^ National Health Commission (NHC) Key Laboratory of Combined Multi-organ Transplantation, The First Affiliated Hospital, School of Medicine, Zhejiang University, Hangzhou, China; ^3^ Institute of Organ Transplantation, Zhejiang University, Hangzhou, China; ^4^ Department of Hepatobiliary and Pancreatic Surgery, Shulan (Hangzhou) Hospital, Hangzhou, China; ^5^ Westlake Laboratory of Life Sciences and Biomedicine, Westlake University, Hangzhou, China

**Keywords:** hepatic, ischemia/reperfusion injury, inflammation, hepatocellular carcinoma, liver transplantation, liver resection

## Abstract

Inflammation is crucial to tumorigenesis and the development of metastasis. Hepatic ischemia/reperfusion injury (IRI) is an unresolved problem in liver resection and transplantation which often establishes and remodels the inflammatory microenvironment in liver. More and more experimental and clinical evidence unmasks the role of hepatic IRI and associated inflammation in promoting the recurrence of hepatocellular carcinoma (HCC). Meanwhile, approaches aimed at alleviating hepatic IRI, such as machine perfusion, regulating the gut-liver axis, and targeting key inflammatory components, have been proved to prevent HCC recurrence. This review article highlights the underlying mechanisms and promising therapeutic strategies to reduce tumor recurrence through alleviating inflammation induced by hepatic IRI.

## Introduction

Hepatocellular carcinoma (HCC) is one of the most prevalent malignancies and the third leading cause of cancer-related mortality in the world (1). Surgical therapies, including hepatectomy and liver transplantation (LT), are the most efficient treatments for patients with HCC (2). However, the prognosis of HCC patients remains dismal due to the high incidence of metastasis and recurrence after surgery, with 5-year recurrence rates reaching up to 70% after liver resection (LR) and 40% after LT (3, 4). Ischemia/reperfusion injury (IRI) refers to the pathophysiological process in which ischemic tissue injury is accentuated following the restoration of blood flow after a period of ischemia (5). Hepatic IRI is an inevitable consequence of LR and LT, which is usually accompanied by intense inflammatory cascade and subsequent damage repair (5). In the mid-19th century, the link between inflammation and cancer was firstly suggested by Rudolf Virchow, based on discovering leukocyte infiltration in neoplastic tissues (6). Nowadays, inflammation has been demonstrated to be strongly associated with tumorigenesis and metastasis of most types of cancer. Targeting inflammation associated with tumor progress has gradually become a critical anti-cancer treatment (7). Currently, multiple preclinical and clinical studies suggest that inflammation induced by hepatic IRI promoted tumor relapse and metastasis after LR or LT (8–12). Meanwhile, alleviating inflammation induced by hepatic IRI is emerging as a promising therapeutic strategy for reducing liver damage and simultaneously suppressing tumor recurrence for HCC patients (13). In this review, we summarize the clinical evidence that hepatic IRI promotes tumor relapse and metastasis. Furthermore, we review recent advances in therapeutic strategies which suppress tumor recurrence through alleviating inflammation induced by hepatic IRI. These avenues of killing two birds with one stone may provide new insights into preventing HCC relapse.

## Hepatic IRI Promotes the Recurrence of HCC: Clinical Evidence in LT

LT, which removes the tumor and the diseased liver at the same time, is a radical treatment modality for HCC. However, tumor relapse is still the most severe threat to the survival of HCC patients after LT (14). Hepatic IRI is a common but thorny problem in different clinical settings such as LT, LR, trauma surgery, and shock. As essential procedures during LT, cold preservation of liver graft and subsequent warm reperfusion when implanted into the recipients result in hepatic IRI, which can be stratified into warm IRI and cold IRI (15). The severity of IRI and subsequent inflammation is positively correlated with ischemia time, which can also be partitioned into warm ischemia time (WIT) and cold ischemia time (CIT) (16).

Clinically, HCC patients with prolonged WIT and CIT are more likely to relapse after transplantation, revealing the links between hepatic IRI and HCC recurrence (8–12). The details of relevant clinical studies are listed in **Table 1**. A retrospective study that enrolled 391 patients found that CIT >10 hours and WIT >50 minutes were associated with significantly increased recurrence (P=0.015 and 0.036, respectively) (8). Another single-center study from Germany also showed that WIT>50 min was an independent risk factor for HCC recurrence (OR=15.5; P <0.001) (9). Orci et al. analyzed the data of 9724 HCC patients from the American Scientific Registry of Transplant Recipients (SRTR). They found that WIT >19 minutes was associated with an increased HCC recurrence risk (P = 0.025) (11). In 2018, our team reached a similar conclusion in HCC patients of China, where hepatitis-B constitutes the main cause of HCC. We recognized CIT >12 hours as the independent donor prognostic factor for predicting HCC recurrence (HR=2.23; P=0.007) (10). In addition to ischemia time, post-reperfusion serum aspartate transaminase (AST) and lactate dehydrogenase (LDH), important indicators reflecting the degree of hepatic inflammatory injury, have also been shown to correlate with the risk of tumor recurrence in patients within the Milan criteria (12).

**Table 1:** The clinical evidence of hepatic IRI promoting recurrence of HCC after LT.

Study	Data sources	N	Underlying liver disease	Milan status	Donor type	Definition of WIT	Definition of CIT	Conclusion
Nagai et al. (8)	Two centers (USA)	391	NA	NA	DBD	Removal of the graft from the cold preservation solution to portal reperfusion	Donor cross-clamping to the removal of the graft from the cold preservation solution	CIT>10 h (HR=1.9; P=0.03) and WIT>50 min (HR=2. 84; P=0.003) were independent risk factors for HCC recurrence
Kornberg et al. (9)	Single center(Germany)	103	Alcoholic(55.3%) Viral (28.2%) Autoimmune(9.7%) Other(6.8%)	In (61.2%) Out (38.8%)	DBD	Removal of the graft from the cold preservation solution to portal reperfusion	In situ cold liver flushing to the removal of the graft from the cold preservation solution	WIT>50 min was an independent risk factor for HCC recurrence (OR=15.5; P <0.001)
Ling et al. (10)	CLTR (China)	673	Hepatitis B cirrhosis(88.9%) Other (11.1%)	In (54.7%) Out (45.3%)	DBD(14.0%) DCD(41.5%) DBCD(44.4%)	Removal of the graft from the cold preservation solution to portal reperfusion	Perfusion of the cold perfusate to the removal of the graft from the cold preservation solution	CIT>12 h was the independent donor prognostic factor for predicting HCC recurrence (HR=2. 234; P=0.007)
Orci et al. (11)	SRTR (USA)	486	NA	NA	DCD	Removal of life support to aortic perfusion with cold preservation solution	In situ aortic cold perfusion to the removal of the graft from the cold preservation solution	WIT>19 min was associated with an increased HCC recurrence risk (HR=4.26; P=0.025)
Grąt et al. (12)	Single center(Poland)	195	Hepatitis C virus infection(67.7%) Hepatitis B virus infection(45.6%)	In (57.9%) Out (42.1%)	DBD	Removal of the graft from the cold preservation solution to portal reperfusion	Clamping of the donor aorta to the removal of the graft from the cold preservation solution	Post-reperfusion AST≥1896 U/L(HR=5.99; P=0.039) and LDH≥4670 U/L (HR=6.08; P=0.04) increased the risk of HCC recurrence after LT in patients within Milan criteria

IRI, ischemia/ reperfusion injury; HCC, hepatocellular carcinoma; LT, liver transplantation; NA, not available; DBD, donation after brain death; DCD, donation after cardiac death; DBCD, donation after brain death followed by circulatory death; WIT, warm ischemia time; CIT, cold ischemia time; HR, hazard ratio; OR, Odds Ratio; CLTR, China Liver Transplant Registry; SRTR, Scientific Registry of Transplant Recipients; AST, aspartate transferase; LDH, lactate dehydrogenase

These findings provide the clinical evidence that inflammation induced by hepatic IRI promoted HCC recurrence and indicate that minimizing ischemia time may be beneficial to alleviating inflammatory injury and reducing the risk of HCC recurrence. However, reducing ischemia time might be difficult in clinical practice, which depends on many factors such as organ allocation system, organ transport, surgery complexity, and experience of transplant teams. Therefore, repair of liver injury or targeting inflammatory mechanisms of hepatic IRI may be more practical solutions in the existing clinical context.

## Inflammation-Targeting Cancer Therapy: Hit Two Birds With One Stone

### Alleviating Hepatic IRI and Subsequent Inflammation From the Source: Machine Perfusion

Currently, cold static storage (CS) is still the most commonly utilized method for liver preservation in LT. Meanwhile, due to the shortage of donor livers, extended criteria donor (ECD) liver grafts such as steatotic grafts, livers from older donors and donors after cardiac death (DCD) are utilized to expand the scarce donor pool (17). These ECD liver grafts are more susceptible to hepatic IRI and are linked to inferior post-transplant outcomes (18). Besides, the use of organs from DCD donors with prolonged WIT and severe steatotic donor livers was proved to increase the risk of HCC recurrence post LT (11, 19). In such a context, the limitations of CS become more obvious. As a future direction of graft preservation, there is increasing attention to machine perfusion (MP) due to its ability to preserve, evaluate and recondition such donor livers prior to transplantation (20).

Oldani et al. found that the use of ischemic rat liver graft accompanied by an increased serum pro-inflammatory cytokine profile increased the risk of cancer recurrence (21). Investigators approximated an *in vivo* normothermic perfusion by reperfusing the liver *in vivo* (animal alive) for two hours before flushing and retrieving. This method attenuated liver injury, measured by the release of liver enzymes (AST and ALT). Besides, prior-to-retrieval reperfusion decreased the recurrence and growth of HCC after transplantation. These effects were partly attributed to the improvement of serum inflammatory cytokine profile. In clinical practice, normothermic extracorporeal membrane oxygenation (NECMO) or NECMO-based normothermic regional perfusion (NRP) is the closest procedure to reproduce the studied model (22). As a technique that allows the *in-situ* perfusion of organs with oxygenated blood in DCD donors, NRP can reduce warm ischemia time and improve graft function (23). This investigation further revealed the possible potential of NRP in preventing hepatic IRI-associated HCC recurrence.

Hypothermic oxygenated perfusion (HOPE) is an emerging organ preservation strategy for marginal grafts (20). The use of artificial, cooled perfusion solutions with oxygenation applied in HOPE has been demonstrated to decrease oxidative stress and inflammatory damage in LT (24). To validate the protective effect of HOPE in HCC recurrence, Mueller et al. compared tumor recurrence rate between HOPE-treated DCD and unperfused DBD liver recipients after liver LT for HCC (25). The results showed that HOPE alleviated general inflammation (measured through plasma CRP of the recipient) and recovered liver functions. He also observed a significantly lower recurrence rate in patients who received end-ischemic HOPE treatment (5.7%, n = 4/70) compared to an unperfused DBD cohort (25.7%, 18/70), despite the use of high-risk DCD grafts (P=0.002). Besides, HOPE treatment improved recurrence-free survival and reduced tumor load significantly compared to an external, nonperfused DCD and DBD liver recipient cohort. HOPE before implantation protected the liver from reperfusion injury and subsequently prevented ongoing tissue inflammation and hypoxia, making the environment less favorable for tumor recurrence and metastasis.

Despite the ability to decrease damage and improve outcomes, hepatic IRI cannot be completely avoided using the techniques above. With the innovations of surgical techniques and NMP, the technique named ischemia-free liver transplantation (IFLT) has been created. Its application is able to procure, preserve, and implant liver grafts from DBD donors without stopping the blood and oxygen supply, thus entirely preventing hepatic IRI (26). A recent propensity-matched cohort study showed that IFLT recipients had a higher RFS rate at 1 and 3 years (92.2% and 86.7%) than conventional LT recipients (88.1% and 53.6%), with much lower liver damage (27). Though the oncological benefit of IFLT needs further observation and validation in a larger sample, this technique shows enormous potential not only for completely avoiding IRI but also for preventing associated HCC recurrence. However, this technique is not applicable to LDLT and DCD LT, which occupy the major types of LT.

### Gut-Liver Axis as a Target in Inflammation-Associated Cancer Recurrence Induced by Hepatic IRI

The close interplay between the intestine and liver in anatomy and function is known as the gut-liver axis. In the liver, two-thirds of total blood flow originates from the portal circulation, which contains gut-derived bacterial products (28). As a “microbial organ”, gut microbiota is involved in the development of many liver disorders through the gut-liver axis (29, 30). During LT and LR, portal vein clamping usually results in venous congestion and hypoperfusion of the intestines, followed by intestinal mucosa damage and increased permeability (31). In these cases, endotoxin/lipopolysaccharide (LPS), a cell wall component of Gram-negative bacteria, could translocate to the portal blood through the impaired intestinal mucosal barrier and intensify hepatic IRI (32).

A recent study has revealed the connection between hepatic IRI and HCC recurrence from a brand-new perspective of the gut-liver axis. The study found that pedicle clamping induced the congestive injury of the bowel wall and subsequent increase of LPS in portal circulation. Upon re-establishment of portal blood flow, bacterial LPS activated the Toll-like receptor 4 (TLR4) signaling pathway in the liver, leading to aggravated IRI, exacerbated inflammatory response, and increased tumor burden (33). In this process, hyperactivation of TLR4 by LPS results in the activation and over-expression of pro-inflammatory transcription factors (TFs) such as nuclear factor-kappaB (NF-κB), activator protein 1 (AP-1) and interferon regulatory factor 3 (IRF3) (34). Increased production of pro-inflammatory cytokines (IL-6, TNF-α, etc.) and chemokines (CXCL1, CXCL10, etc.) intensify hepatic IRI and upregulate the components involved in the progression and metastasis of HCC including angiogenesis cytokines and immunosuppressive cells such as Tregs (35).

**Figure 1:**
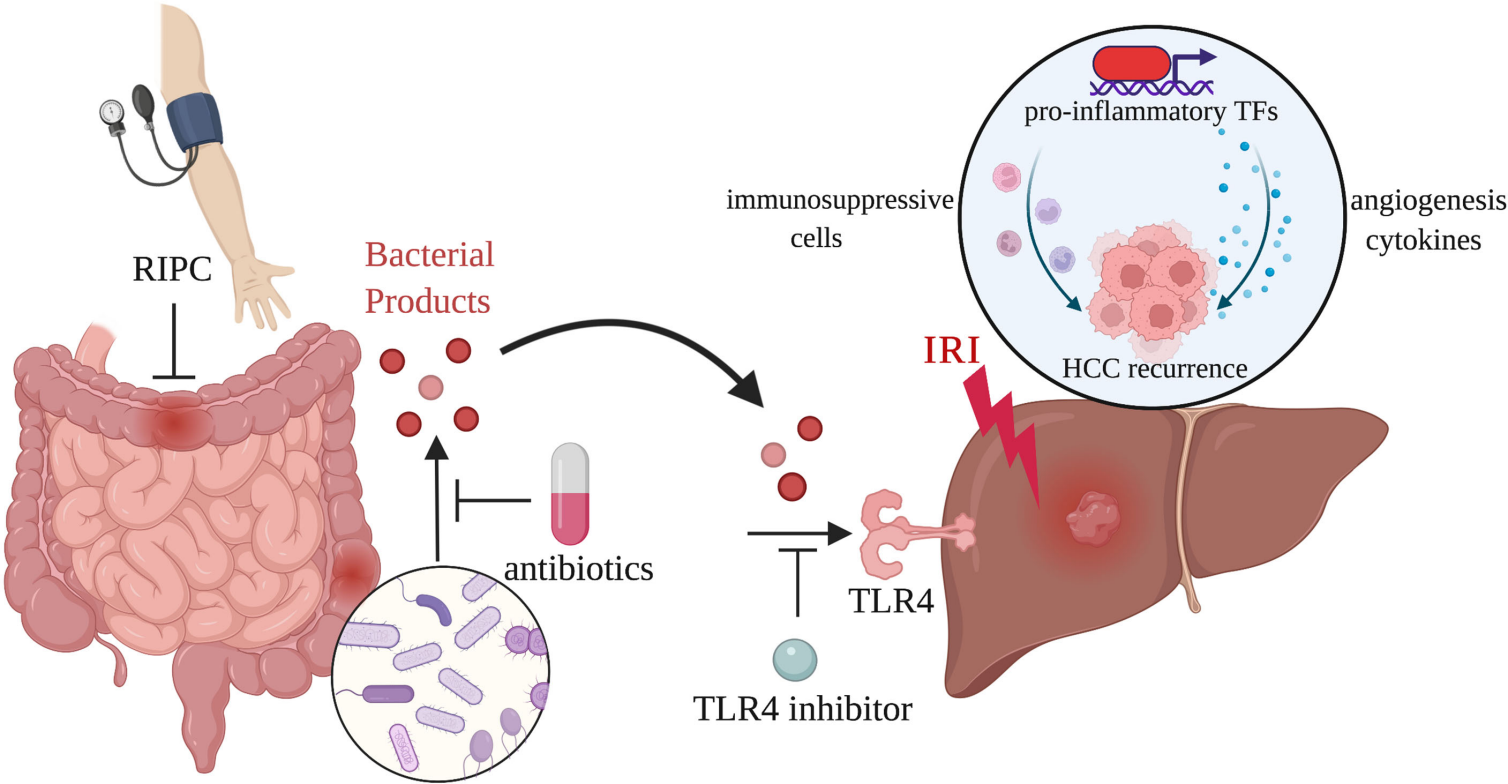
The gut-liver axis as a target in inflammation-associated cancer recurrence induced by hepatic IRI. Portal vein clamping during LT and LR leads to venous congestion and hypoperfusion of the intestines. Bacterial products released through damaged intestinal mucosa activate TLR4 signaling pathway in the liver, resulting in the exacerbated inflammatory response and increased tumor burden. RIPC, antibiotics, and TLR4 inhibition are able to act on the gut-liver axis to reduce inflammation-associated HCC recurrence.

The researchers also explored several therapeutic approaches to suppress tumor growth by targeting the gut–liver axis (33). They found that remote ischemic preconditioning (RIPC) induced by brief and repeated sequences of femoral vascular bundle clamping and declamping could prevent liver injury and associated HCC recurrence through dampening small bowel venous ischemia and preventing bacterial translocation. Despite promising animal evidence supporting the protective effect of RIPC on hepatic IRI, the results of several clinical trials are inconsistent, indicating that RIPC’s clinical role remains to be further confirmed (36–39). Besides RIPC, the study also showed that gut decontamination (antibiotics) and pharmacological TLR4 inhibition confer systemic protection against inflammation-mediated accelerated HCC growth. Their results indicated that modulating the gut–liver axis during liver surgery could be a potential target in combating HCC recurrence. Predictably, gut microecological therapy will gradually become an emerging treatment modality in preventing inflammation-induced HCC recurrence. **Figure 1** displays the schematic diagram of the gut-liver axis as a target in inflammation-associated cancer recurrence induced by hepatic IRI.

### Targeting Key Inflammatory Components in the Tumor Microenvironment

Hepatic IRI is a dynamic process including two related stages: ischemic injury and inflammation-mediated reperfusion injury (40). In the phase of ischemia, hepatocytes are exposed to oxygen deprivation, ATP depletion, and pH decreasement (41). These changes result in the accumulation of reactive oxygen species (ROS), increasement of intracellular calcium concentrations which lead to hepatocyte damage and different cell death programs such as apoptosis, necrosis, ferroptosis and pyroptosis (13, 42). The following reperfusion triggers inflammatory cascade that aggravates hepatocyte injury through multiple mechanisms. In addition to cell death programs and metabolic disorders, innate immune activation plays a major role in this process (5). After sensing damage-associated molecular patterns (DAMPs) released from damaged or dead cells, Kupffer cells become activated through pattern-recognition receptors (PRRs) and release chemokines and cytokines to initiate a pro-inflammatory response (43).

The inflammatory cascade after reperfusion involves multiple inflammatory components and their interactions. Activated Kupffer cells release cytokines like IL-1β and chemokines such as CXCL1 and CXCL2 to activate and recruit neutrophils in damaged site (44). Platelets activated by DAMPs also serve as immune mediators which express CXCL4 and P-selectin and promote the recruitment of monocytes and neutrophils to the inflammation site (45–47). The expression of NKG2D (an activating receptor for NK cells) is significantly upregulated in the reperfused liver (48). Increased NK cells recruited to the liver can not only exacerbate IRI by producing IFNγ but also increase the production of IL-17, which promotes the recruitment of neutrophils (49, 50). Hepatic IRI also elicits a robust adaptive immune response in which CD4+ T cells mediate aggravated inflammatory damage (40).

The balance of pro-inflammatory and anti-inflammatory components determines the outcome of inflammation during IRI (44). Multiple clinical and experimental studies have suggested that intense and continuous inflammation induced by hepatic IRI promoted tumor recurrence *via* activating cellular signaling for tumor cell proliferation, adhesion, invasion and angiogenesis (8, 51). Hypoxia-inducible factor 1α (HIF1α), matrix metalloproteinase 9 (MMP9), vascular endothelial growth factor (VEGF) and other key molecules play important roles in this process (52).

After the initiation of inflammation, some anti-inflammatory components and repair factors will be recruited into the inflammatory microenvironment to inhibit the progress of inflammation and promote injury repair. Anti-inflammatory cytokines, such as IL-4 or IL-13, and apoptotic cells promote *in situ* macrophage polarization and reprogramming and then initiate the resolution phase of inflammation (53–55). Some anti-inflammatory neutrophil subtypes exert anti-inflammatory and repair functions through inhibiting cytotoxic T cells (CTLs), promoting macrophage polarization and angiogenesis (44, 56). Besides, regulatory T cells (Tregs), myeloid-derived suppressor cells (MDSCs), endothelial progenitor cells (EPCs), platelets and many other factors also play critical roles in regulating inflammation and its resolution (44, 57, 58).

**Figure 2:**
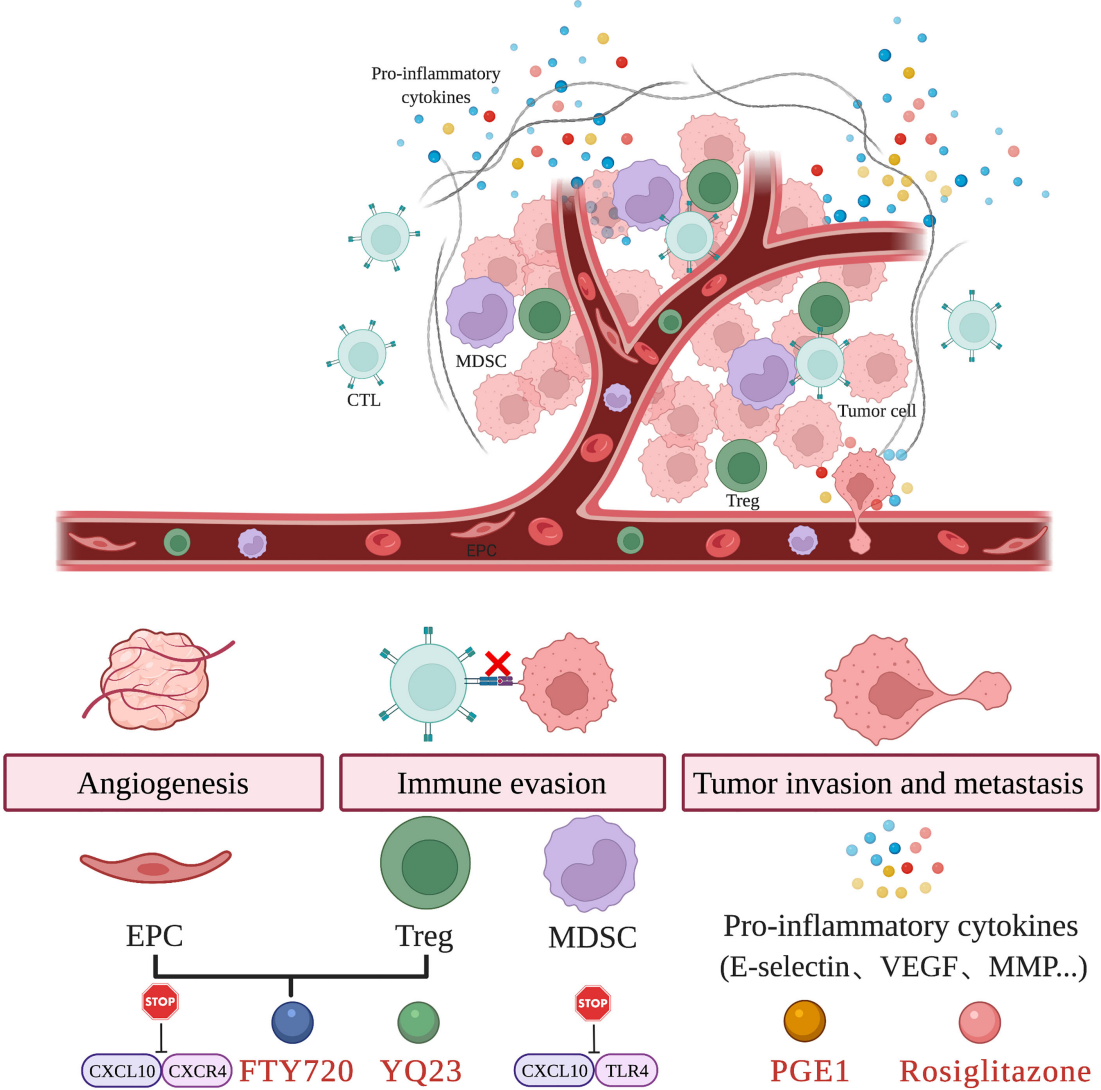
Targeting key inflammatory components in the tumor microenvironment. Hepatic IRI results in the recruitment of EPC, Treg, and MDSC as well as the release of pro-inflammatory cytokines. These changes in the tumor microenvironment induce tumor angiogenesis, immune evasion and promote tumor invasion and metastasis. The use of agents such as FTY720, YQ23, PGE1, Rosiglitazone and targeting key inflammatory pathways are able to attenuate hepatic IRI and prevent tumor recurrence.

However, in the setting of oncology, these beneficial components may also result in worsened oncologic outcomes. Some components responding to inflammation have been proved to promote HCC recurrence through different mechanisms, including mediating the immune escape and angiogenesis of tumor as well as causing tumor cells to be more aggressive by triggering tumor cell adhesion, migration, and invasion (13). Targeting key inflammatory components in the tumor microenvironment may suppress tumor recurrence. Compared to machine perfusion, this method provides more targeted treatment for HCC patients and can be applied to both LR and LT. **Figure 2** displays the key inflammatory components in the tumor microenvironment and relevant therapies.

Immune escape is a crucial characteristic during tumor growth and metastasis. MDSCs and Tregs have been identified as key immune cell subsets that mediate tumor immune escape (59). Of them, MDSCs are a heterogeneous population of immunosuppressive cells derived from myeloid progenitors, which can be upregulated by inflammatory mediators such as IL-2, GCSF, and so on (57). MDSCs suppress the immune system by various mechanisms including (i) inhibiting CTLs proliferation and activation by increased nitric oxide (NO), nitrotyrosine and ROS secretion, and decreased l-arginine production (ii) inducing immunosuppressive cells like T helper (Th) 17 cells and Tregs (60); Tregs are crucial suppressors of immune responses and are essential for maintaining immunological tolerance and controlling inflammatory diseases (61, 62). In the process of tumor immune escape, Tregs can suppress antigen presentation by DCs, CD4+ Th cell function and promote intratumoral T cell exhaustion through secreting TGF-β, IL-10 and IL-35 (63, 64). Recent findings also demonstrated the crosstalk between Tregs and MDSCs, which found that Tregs regulate MDSCs differentiation and function through TGF-β and the programmed death ligand 1 (B7-H1) (65, 66). In HCC patients, MDSCs and Tregs were found to be significantly elevated in the peripheral blood and tumor (67, 68). Besides, emerging tumor immunotherapy targeting MDSC and Tregs has effectively inhibited the progression of HCC (63, 69).

In addition to immune escape, tumor angiogenesis also plays a key role in tumor recurrence. EPCs are a kind of vascular progenitor with high proliferative potential that are derived from bone marrow (70). Inflammatory endothelial injury can trigger mobilization, homing, and transdifferentiation of EPCs, thereby contributing to the repair of injured endothelium (71). Recent studies have found that EPCs participate in neovascularization during acute ischemic injury (58). However, mobilization of EPCs also occurs in response to low oxygenation during tumor growth (72). They are able to facilitate the release of proangiogenic cytokines and promote vessel incorporation and stabilization (73). Multiple studies have shown that mobilization of circulating EPC results in tumor neovascularization and accelerates tumor growth and metastasis (74, 75). Besides, the inhibition of EPCs mobilization restrained angiogenesis and tumor progression (76).

In the inflammatory cascade triggered by hepatic IRI, chemokines and chemokine receptors are involved in the recruitment of inflammation-responsive components discussed above (77). Selectively targeting these pathways has been heralded as a promising avenue for suppressing these cell subsets. Through a series of clinical analyses, *in vitro* and *in vivo* experiments, Liu et al. found that MDSCs recruited by CXCL10/TLR4 during acute phase inflammation played a critical role in tumor recurrence after LT. Targeting MDSC mobilization *via* CXCL10/TLR4 signaling could not only protect the liver graft from IRI but also reduce tumor recurrence after transplantation (78). Ling et al. identified that post-transplant enhanced CXCL10/CXCR3 signaling in small-for-size liver grafts directly induced EPC mobilization, differentiation, and neovessel formation, which further promotes tumor growth (79). Besides, CXCL10/CXCR3 signaling upregulated at liver graft injury directly induced the mobilization and intragraft recruitment of Tregs, which further promoted HCC recurrence after transplantation (80). Targeting CXCL10/CXCR3 signaling inhibited the mobilization of Treg and EPC, attenuated early-phase liver graft injury, and prevented late-phase tumor recurrence/metastasis after transplantation (79, 80).

In addition to genetic approaches and pharmacological inhibitors, some drugs can also act through the above pathways. FTY720 (fingolimod) is a sphingosine-1-phosphate (S1P) receptor agonist that has been approved by FDA as a treatment for multiple sclerosis (MS) (81). In models of lung, kidney, and liver IRI, FTY720 unfolds a demonstrated anti-inflammatory effect (82–84). Furthermore, FTY720 has exhibited a strong anti-tumor activity in liver cancer, breast cancer, bladder cancer, and so on (85–88). Li et al. found that FTY720 significantly attenuated hepatic IRI and tumor metastasis after LR through the downregulating CXCL10/CXCR3 signaling pathway. In their study, FTY720 treatment reduced the population of circulating EPCs and Tregs and thereby limited tumor angiogenesis and enhanced antitumor immune response (89, 90). A novel oxygen carrier called YQ23 played a similar role in suppressing liver tumor metastasis after major hepatectomy and partial hepatic I/R injury through increasing liver oxygenation and reducing the number of circulating EPCs and Tregs (91).

A number of studies have indicated that proinflammatory cytokines such as E-selectin, VEGF and MMP activated by hepatic IRI promoted tumor invasion and metastasis (92–94). Ligands of PPARγ, such as rosiglitazone can downregulate the expression of proinflammatory cytokines that are associated with tumor metastases through inhibiting the NF-κB signaling pathway (95–97). In an experimental mouse model of hepatic IRI-induced HCC metastasis, rosiglitazone exerts a protective effect on hepatic IRI and significantly inhibits tumor metastasis following that. As reported in this article, the dual action may be attributed to inhibited NF-κB signaling and reduced expression of proinflammatory cytokines in liver (98).

Prostaglandins (PGs) are products of arachidonic acid metabolism *via* the cyclooxygenase pathway, which are produced primarily by activated Kupfer cells during hepatic IRI (99). PGs exert anti-inflammatory effects by prevention of leucocyte migration and down-regulation of pro-inflammatory cytokines (100). The administration of alprostadil, a synthetic stable form of prostaglandin E1(PGE1), was shown to attenuate hepatic IRI and improve liver graft function (101). In a retrospective clinical study, Kornberg et al. found that treating hepatic IRI with alprostadil reduced systemic inflammation levels and the risk of early HCC recurrence following LT (102).

Besides these mentioned strategies above, glutathione peroxidase 3, thymoquinone, and zinc finger protein A20 also show the potential to be “one stone for two birds” strategies that attenuate hepatic IR injury and prevent tumor recurrence after liver surgery (103–105). However, their inhibition of hepatic IRI-associated recurrence needs further experimental research and clinical verification.

## Conclusion and Prospect

Collectively, the inflammation induced by hepatic IRI plays a crucial role in the development and progression of HCC, which contributes to several hallmarks of HCC, such as promoting proliferative and survival signaling, inducing angiogenesis, evading immune surveillance, and activating invasion and metastasis. Besides, alleviating inflammation such as machine perfusion, regulating the gut-liver axis and targeting key inflammatory components or inflammatory pathways is believed to be an attractive therapeutic strategy. However, current evidence mainly comes from animal models. More clinical studies with larger numbers of patients will be required. Moreover, not all measures are applicable to LT or LR due to the differences in surgical procedures, use of immunosuppressive agents and postoperative management. Additional validation is needed in different models and patient populations. In the future, we advocate using advanced techniques such as single-cell multi-omics and spatial omics to explore the mechanism and targets for therapeutic intervention comprehensively. Meanwhile, we believe that combining different approaches at different time points should yield better outcomes for patients.

## Author Contributions

XX and DL designed and supervised the study. HC and XY performed the article searching and wrote the manuscript. ZH, CH, HL, ZL and MY revised the manuscript. All authors contributed to the article and approved the submitted version.

## Funding

This work was supported by grants from The Major Research Plan of the National Natural Science Foundation of China (92159202), Key Program, National Natural Science Foundation of China (81930016), National Key Research and Development Program of China (2021YFA1100500), Key Research & Development Plan of Zhejiang Province (No. 2019C03050), and Young Program of National Natural Science Funds (No. 82000617).

## Conflict of Interest

The authors declare that the research was conducted in the absence of any commercial or financial relationships that could be construed as a potential conflict of interest.

## Publisher’s Note

All claims expressed in this article are solely those of the authors and do not necessarily represent those of their affiliated organizations, or those of the publisher, the editors and the reviewers. Any product that may be evaluated in this article, or claim that may be made by its manufacturer, is not guaranteed or endorsed by the publisher.
